# Activity of Lysozyme Against Multidrug-Resistant *Salmonella* Heidelberg and *Salmonella* Minnesota Isolated from Broilers

**DOI:** 10.3390/ani16010019

**Published:** 2025-12-20

**Authors:** Leticia Soares Franco, Marcos Paulo Vieira Cunha, Carina Megumi Nishio, Reinaldo Kanji Kato, Fernanda Borges Barbosa, Vasco Túlio Moura Gomes, Monique Ribeiro Tiba Casas, Andrea Micke Moreno, Terezinha Knöbl

**Affiliations:** 1 School of Veterinary Medicine and Animal Science, University of São Paulo, São Paulo 05508-270, Brazil; leticiasf91@gmail.com (L.S.F.); morenoam@usp.br (A.M.M.); 2 Biogenic Group Indústria e Comércio Ltda, Taboão da Serra 06790-030, Brazil; 3 Adolfo Lutz Institut, São Paulo 01246-000, Brazil

**Keywords:** poultry production, antimicrobial resistance, avian diseases, enzyme-based alternatives

## Abstract

Bacterial resistance in foodborne pathogens is a global concern and has stimulated the search for alternative compounds to antimicrobials. In this context, the prevention of colonization by *Salmonella* spp. in poultry production is particularly important. This study investigated the bactericidal effect of lysozyme on *Salmonella* Heidelberg and *Salmonella* Minnesota. A total of 44 serotyped isolates were subjected to minimum inhibitory concentration (MIC) testing against 17 distinct antibiotics. Subsequently, the same isolates were subjected to minimal bactericidal concentration (MBC) with lysozyme at concentrations ranging from 15 to 2000 ppm. One strain of *S. Heidelberg* was selected for an in vivo challenge. Seventy-two male chicks were randomly divided into three experimental groups, and two of them were challenged on the second day with 0.5 mL of an inoculum containing 1 × 10^5^ CFU/mL. One of these groups was treated with lysozyme at a concentration of 1000 ppm per bird for 21 days. MIC tests showed that the multidrug resistance rate was 97.72%, with susceptibility only to fosfomycin, florfenicol, and meropenem. After the in vitro exposure of these isolates to lysozyme, 86.36% were inhibited at concentrations ≤ 15 ppm. The in vivo tests showed a significant reduction in the total number of chickens colonized by *S. Heidelberg* at 2, 5, 7, 14, 18, and 21 days of farming. On the day of slaughter, the percentage of positive birds in the inoculated group was 63.63%, while that in the group treated with lysozyme was 26.08%. These data highlight the potential use of lysozyme as an alternative to antibiotics in poultry production.

## Simple Summary

The presence of *Salmonella* in food can result in diseases, including diarrhea and fever. The consumption of chicken meat contaminated with the bacteria is a concern worldwide. Therefore, there is great interest in finding alternatives to antibiotics to keep poultry healthy and prevent food contamination. In this study, we tested whether lysozyme, a natural enzyme, could kill *Salmonella*, a common bacterium in chicken production in Brazil. We analyzed 44 *Salmonella* isolates from poultry feces in four Brazilian states. The two most common variants were *Salmonella* Heidelberg and *Salmonella* Minnesota, both resistant to several antibiotics. In the laboratory, we found that small amounts of lysozyme were sufficient to kill most of these bacteria. We then tested the product on infected chickens and observed that those treated with lysozyme had less *Salmonella* in their intestines after 21 days. These results show that lysozyme can help reduce the presence of *Salmonella* in poultry, indicating that it may be a promising alternative to antibiotics in chicken production.

## 1. Introduction

*Salmonella enterica*, a member of Enterobacteriaceae family, is a global health threat, associated with approximately 11 to 20 million cases of foodborne disease per year, with 155,000 deaths [1].

Multidrug-resistant *Salmonella* represents a significant and escalating global health threat linked to antimicrobial resistance (AMR). According to Wang et al. (2022), the number of antibiotic-resistant genes per non-human origin *Salmonella* increased 2.69 times in 14 years [2]. The increasing incidence of AMR in *Salmonella* has become a pressing concern, particularly as strains have exhibited resistance to several common classes of antibiotics, as beta-lactams and quinolones [2].

Recent reports have described the factors driving antimicrobial resistance in *Salmonella*, highlighting trends of increasing multidrug resistance, particularly in developing countries [3,4]. Wang et al. (2025) analyzed 208.233 genomes of *Salmonella* spp. from 148 countries between 1900 and 2023 [3]. The results showed that 99.94% of serovar *S.* Heidelberg were MDR, with resistance to quinolone (99.98%), fosfomycin (99.08%), and beta-lactams (35.55%) [3]. Fluorquinolone-resistant, third-and-fourth generation cefalosporins *Salmonellas* have been categorized as high-priority and critical-priority pathogens, respectively, since 2024 [4].

Currently, Brazil is the second-largest producer of chicken meat and the largest exporter in the world, producing more than 14.8 billion tons of chicken, with significant surpluses exported to over 150 countries. Therefore, the prevention of broiler colonization by *Salmonella* is considered a high priority on Brazilian poultry farms. The temporal dynamics of *Salmonella* spp. in Brazil revealed a significant reduction in serovar *S.* Enteritidis after the introduction of vaccines; however, the serovars *S.* Minnesota and *S.* Heidelberg have emerged and have acquired resistance genes associated with beta-lactams, fosfomycin, and quinolone, as well as disinfectants [5].

Following the restriction on antibiotic growth promoters (AGPs) as feed additives in 2020, several alternative products, such as prebiotics, probiotics, essential oils, immunostimulants, and enzymes, have been employed to inhibit enteric bacteria [6]. Strict biosafety, intensive surveillance measures, and the use of live vaccines and alternative additives have not been sufficient to prevent paratyphoid serovars in Brazilian poultry farms, particularly those involving β-lactamase-producing strains of *S*. Heidelberg (*S*H) and *S.* Minnesota (*S*M) [5,6,7].

Lysozyme or 1,4-β-N-acetylmuramidase is a natural enzyme that plays an important role in host defense, with bacteriostatic and bactericidal activity against Gram-positive microorganisms [8,9]. Lysozymes are present in body fluids such as tears, saliva, milk, colostrum, and egg albumin. Its enzymatic action occurs through binding between N-acetylmuramic acid and N-acetylglucosamine in peptidoglycan, leading to lysis of the bacterial wall [8,9].

Lysozyme can be commercially extracted and used as a natural food preservative against biofilm formation by various pathogenic bacteria [10]. Under certain conditions, lysozyme can be modified by industrial processing techniques, such as fermentation, to extend its activity against Gram-negative bacteria [8,9,10,11]. Although the bactericidal effect of lysozyme is well recognized, the data in the literature remain insufficient to evaluate whether this compound is a viable alternative for controlling strains of MDR *Salmonella* on poultry farms.

The aim of this study was to investigate the bactericidal effects of lysozyme against *S. Heidelberg* and *S. Minnesota* from broiler chickens in Brazil.

## 2. Materials and Methods

### 2.1. Bacterial Isolates

A total of 44 isolates of *Salmonella* spp. were cultured from fecal samples of broiler chickens, between 2017 and 2022, from four different farms located in three different Brazilian states. Samples were cultured in peptone water, pre-enriched with sodium tetrathionate, and plated on XLT4 and CHROMagar™ *Salmonella* (Difco, Detroit, MI, USA), with incubation at 37 °C for 24–48 h. Colonies of typical *Salmonella* morphology were subjected to Polymerase Chain Reaction (PCR) to confirm identification, following the protocols described by Alvarez et al. [12].

These isolates belong to the Enterobacteriaceae collection of the Avian Medicine Laboratory of the School of Veterinary Medicine and Animal Science of the University of São Paulo (FMVZ-USP). This project was approved by the Animal Use Ethics Committee (CEUA) of FMVZ-USP (CEUA 8036250718).

### 2.2. Serogroup Determination

The isolates were sent to the Adolfo Lutz Institute Laboratory (IAL), São Paulo, a National Reference Laboratory for *Salmonella*, for rapid serum agglutination testing according to the Kauffman–White scheme [13]. Some isolates not typed using this methodology underwent in silico analysis after whole genome sequencing (WGS). Six isolates representative of the identified serogroups were grown at 37 °C for 24 h on MacConkey agar. Genomic DNA extraction was performed using the DNeasy kit (Qiagen, Redwood City, CA, USA). A NanoDrop spectrophotometer was used to quantify DNA concentrations. Genomic libraries were prepared using the Nextera XT kit (Illumina, San Diego, CA, USA), and next-generation sequencing was performed on the Illumina MiSeq Platform (Illumina, CA, USA). Genomes were assembled using SPAdes v.3.10.1 [14]. Contigs <200 bp and <10-fold coverage was excluded from downstream analyses [15,16]. In silico analysis was performed with the NASP tool v1.0 [17].

### 2.3. Minimum Inhibitory Concentration

The isolates were subjected to the minimum inhibitory concentration (MIC) test, according to the plate microdilution method, following the Clinical and Laboratory Standards Institute (CLSI) documents M100, M31-A3, and VET01 [18,19,20]. The antimicrobials tested were amoxicillin with clavulanic acid (0.5–64 mg/L), nalidixic acid (8–128 mg/L), ampicillin (1–64 mg/mL), azithromycin (4–64 mg/L), ceftiofur (0.25–8 mg/L), ciprofloxacin (0.06–8 mg/L), chloramphenicol (4–64 mg/L), colistin (1–16 mg/L), florfenicol (0.5–8 mg/L), fosfomycin (8–512 mg/L), gentamicin (0.5–32 mg/L), marbofloxacin (0.12–8 mg/L) meropenem (0.25–8 mg/L), neomycin (4–16 mg/mL), oxytetracycline (2–32 mg/L), trimethoprim–sulfamethoxazole (2–76 mg/L), and sulfonamide (256–1024 mg/L).

To prepare the inoculum, *Salmonella* spp. strains were grown in brain–heart infusion (BHI) broth (Difco, Detroit, USA) and incubated at 37 °C for 24 h. The turbidity of the culture was adjusted with sterile saline solution (0.9%) to achieve a density equivalent to the 0.5 McFarland standard, confirmed spectrophotometrically (OD600 = 0.150). After adjustment, the bacterial suspension was diluted 1:1000 in Mueller–Hinton II broth (Difco, Detroit, USA), obtaining a final concentration of approximately 5 × 10^5^ CFU/mL. Then, 50 µL of the inoculum was dispensed into each well of the microplate, sealed with sterile adhesive, and incubated at 37 °C for 24 h.

*Escherichia coli* ATCC 25922 and *Staphylococcus aureus* ATCC 25923 were used as quality control strains. The multidrug resistance was classified as described by Schwarz et al. 2010 [21].

### 2.4. Minimum Bactericidal Concentration of Lysozyme

To determine the Minimal Bactericidal Concentration of lysozyme against *Salmonella* strains, an inoculum suspension with 1 × 10^6^ cells/mL of each isolate was prepared.

The test was performed in triplicate, using a lysozyme^®^ from Zhejiang Aegis Biotech Co., Ltd. (Aegis Group, Hangzhou, China). The solution was prepared in salt-free LB broth (Difco, Detroit, USA), pH 4.31. In 96-well microplates, lysozyme was diluted from an initial concentration of 2000 µg/mL, followed by serial dilutions 1000, 500, 250, 125, 62, 31, and 15 µg/mL. After this, 10 μL of inoculum were added in each well of the plates containing LB broth and subcultured onto LB agar plates, using a sterile Scienceware^®^ plate replicator (Wayne, NJ, USA). Colony growth was assessed after 24 h of incubation at 37 °C. The interpretation criteria of MBC revealed the lowest concentration, where no growth or ≤3 colonies were observed on agar (≤0.1% of survivor’s cells). A strain of *Staphylococcus aureus* ATCC 29213 was used as a quality control strain on each test plate.

### 2.5. In Vivo Lysozyme Activity Against Intestinal Colonization by Salmonella Heidelberg in Broiler Chickens

The in vivo experimental protocol was approved by the CEUA of FMVZ-USP (CEUA 7091130921) and conducted at the Center for Avian Pathology (CEPA-VPT-FMVZ-USP), over three weeks. The number of birds was defined as the smallest sample size necessary to identify statistical differences between groups, in accordance with the requirement to reduce the use of animals under current Brazilian regulations of CONCEA (National Council for the Control of Animal Experimentation).

A previously selected multidrug-resistant strain of *Salmonella* Heidelberg (SH) was used in the in vivo test (GenBank/OneBr accession number JABFEH000000000). The strain was selected because it contains the following resistance genes: *aac(6′)-Iaa*, *aac(3)*-VIa, *ant(3″)-Ia*, gyrA*^1^, parC*^2^, *sul1*, *sul2*, *tet*(A), *bla*_CTX-M-2_, *fosA7*, *qacE*. The strain was cultured in BHI broth at 37 °C for 18 h. The inoculum was standardized to a concentration of 1 × 10^5^ CFU/mL in phosphate-buffered saline (PBS, pH 7.2) and refrigerated until inoculation.

Seventy-two male Cobb chicks, purchased from a hatchery and housed on the first day of life, were used. All animals were evaluated for *Salmonella* spp. presence in their meconium before the start of the experiment. The birds were randomly distributed into 1.2 m^2^ pens and received antibiotic free commercial feed and drinking water ad libitum. Biosecurity measures were adopted to prevent horizontal transmission between groups.

The experimental design was completely randomized, with three groups and three replicates, totaling eight birds per replicate. T1 (negative control group) was inoculated via gavage with 0.5 mL of PBS on day 2 of life, without lysozyme administration. T2 (positive control group) was inoculated via gavage with 0.5 mL of *S*H suspension on day 2 of life, without lysozyme administration. T3 (treated group) was inoculated via gavage with 0.5 mL of *S*H suspension on day 2 of life. From day 3 onwards, birds in group T3 received 0.5 mL of lysozyme solution (1000 ppm) daily, also by gavage, for three weeks.

On days 2, 5, 7, 14, and 18 of life, cloacal swabs were collected from four birds per replicate in all experimental groups for detection of *S*H.

Analyses were performed in accordance with the Ministry of Agriculture and Livestock (MAPA) Normative Instruction No. 126/1995 [22]. Fecal samples were diluted in 1% buffered peptone water, incubated at 37 °C for 24 h, enriched in Tetrathionate broth (Difco, Detroit, USA) (1:20), incubated at the same conditions, and subsequently plated on XLT4 agar (Difco, Detroit, USA), incubated under the same conditions. Samples with colonies of typical *Salmonella* morphology were subjected to PCR confirmation using insulated isothermal PCR (Pockit™ Central—Gene Reach Biotechnology Corp., Taiwan, China).

On day 21 of life, all birds were euthanized for *S*H quantification in the ingluvium and cecum. The organs were aseptically removed, weighed, and macerated in sterile bags at a 1:10 dilution in peptone water (initial dilution 10^−1^). From this suspension, serial dilutions up to 10^−8^ in PBS were prepared, and 100 μL of each dilution was plated in duplicate on XLT4 agar and incubated at 37 °C for 24 to 48 h for colony counting.

### 2.6. Statistical Analysis

In the in vivo challenge, data for statistical analysis were compiled into two databases. The first included the frequency of positive or negative animals on days 2, 5, 7, 14, and 18 based on microbiological evaluations. The second contained data on bacterial counts and the frequency of positivity on day 21, analyzed using both nominal qualitative and continuous quantitative approaches.

All statistical analyses were performed using a two-tailed significance level (α = 0.05) and a 95% confidence interval (CI), with computational support from R software (2022) [23] and IBM SPSS Statistics for Windows, version 25 (IBM Corp., Armonk, NY, USA).

Qualitative variables were described using frequencies and their respective confidence intervals, while quantitative variables were summarized using measures of central tendency (mean and median) and measures of dispersion (standard deviation, interquartile range, minimum, and maximum).

Associations between qualitative variables were assessed using the chi-square test with Yates’ correction. The relationship between quantitative variables and treatment groups was evaluated using the Kruskal–Wallis test, followed by Dunn’s post hoc test when statistically significant differences were found.

The association between treatment groups and the temporal trend of animal positivity was assessed using a non-parametric ANOVA for longitudinal data [24], and implemented in the nparLD package [25].

## 3. Results

### 3.1. Serogroup Identification

Of the 44 isolates submitted for serogroup determination, 28 (64%) were identified as *S*. Heidelberg (*S*H) and 16 (36%) as *S*. Minnesota (*S*M).

### 3.2. MIC

MIC results revealed resistance to different classes of antimicrobials (Table 1). *Salmonella* spp. strains exhibited 63.6% (*n* = 28) resistance to nalidixic acid; 56.8% (*n* = 25) resistance to amoxicillin with clavulanic acid; 75% (*n* = 33) resistance to ampicillin; 25% (*n* = 11) resistance to azithromycin; 36.6% (*n* = 16) resistance to ceftiofur; 59% (*n* = 26) resistance to ciprofloxacin; 4.5% (*n* = 2) resistance to chloramphenicol; 18.1% (*n* = 8) resistance to gentamicin; 2.2% (*n* = 1) resistance to marbofloxacin; 34% (*n* = 15) resistance to neomycin; 95.4% (*n* = 42) resistance to oxytetracycline; and 97.7% (*n* = 43) resistance to sulfonamides. The highest resistance rates were observed for sulfonamides and oxytetracycline at 97.7% and 95.4%, respectively. However, the combination of sulfamethoxazole with trimethoprim resulted in 100% susceptibility. No resistance was observed to fosfomycin, florfenicol, or meropenem.

In the beta-lactam group, the strains showed 75% (*n* = 33) resistance to ampicillin and 56.8% (*n* = 25) to amoxicillin with clavulanate, both with a MIC90 value of 64 μg/mL. For ceftiofur, the strains showed 36.6% (*n* = 16) resistance, with MIC50s and MIC90s values between 2 μg/mL and 8 μg/mL, respectively. In the quinolone group, a total of 63.6% (*n* = 28) of the isolates showed resistance to nalidixic acid, and 59% (*n* = 26) of these isolates were also resistant to ciprofloxacin, with MIC50s and MIC90s values of 1 and 2 μg/mL, respectively. However, marbofloxacin demonstrated better antimicrobial activity, with only 2.2% (*n* = 1) of the isolates classified as resistant. For the aminoglycosides tested, neomycin showed 34% (*n =* 15) resistance, while gentamicin showed 18.1% (*n =* 8). The MIC50 and MIC90 ranged from 8 to 16 μg/mL for neomycin and from 16 to 32 μg/mL for gentamicin. Resistance rates for the macrolide azithromycin were 25% (*n* = 11), with an MIC90 of 32 μg/mL. Lower resistance rates were observed for colistin and chloramphenicol, both at 4.5% (*n* = 2).

The results showed that 97.72% (*n* = 43) of the isolates were multidrug-resistant (MDR), defined as resistance to three or more distinct classes of antibiotics. Only one isolate was resistant to two classes of antimicrobials (sulfonamides and quinolones).

### 3.3. Lysozyme Microdilution Test

In the lysozyme microdilution test, 86.36% (*n* = 38/44) of the isolates showed growth inhibition at concentrations ≤ 15 ppm, 2.2% (*n* = 1/44) at 31 ppm and 1000 ppm, and 4.5% (*n* = 2/44) at 250 ppm and >2000 ppm. The MBC50% and MBC90% were ≤15 ppm for *S*H and *S*M isolates in all integrations evaluated (Table 2).

### 3.4. In Vivo Lysozyme Activity Against Intestinal Colonization by Salmonella Heidelberg in Broiler Chickens

All cultures performed prior to bird inoculation yielded negative results, confirming that the animals were free of *Salmonella* spp. at the beginning of the experiment. Microbiological evaluation results from fecal samples collected on days 2, 5, 7, 14, 18, and 21 are summarized in Table 3. All birds in the negative control group remained free of *Salmonella* spp. throughout the study period. During the final week of the experiment, six birds died—three from the negative control group, two from the positive control group, and one from the treatment group. In all cases, ascites was identified as the cause of death.

Regarding in vivo lysozyme activity against *S*H, the birds in the positive control group showed a positivity rate of 41.66% (*n* = 5) on the day of inoculation, reaching a maximum of 66.66% (*n* = 8) on days 5 and 14 (Table 3). At slaughter, the percentage of positive birds was 63.63% (*n* = 14). The lysozyme-treated group showed a lower number of colonized birds throughout the experimental period, with a percentage of 16.6% (*n* = 2) on the day of inoculation, reaching a maximum of 58.33% (*n* = 7) on day 14. On the day of slaughter, the percentage of positive birds was 26.08% (*n* = 6), representing a 37.55% reduction in the number of colonized birds.

Descriptive statistical analysis of the frequency of *S. Heidelberg* positivity is presented in Table 4. The negative control group was excluded from the analysis due to the absence of data variability.

The results in Table 4 show a statistically significant difference between the lysozyme-treated group and the positive control group (*p* = 0.025). Although large variations in isolation frequency were observed among the other time points, the analysis applied did not reveal statistically significant differences.

To increase statistical power and enable a longitudinal assessment, a non-parametric ANOVA test was performed. This analysis identified significant differences in isolation frequencies between the control and treatment groups, while no significant differences were found across the evaluation time points (Table 5).

After treatment, no birds showed colonization in the ingluvium (Table 6). In the positive control group, values ranging from 1 × 10^2^ to 1 × 10^5^ CFU/g were detected, with an average of 1.4 × 10^3^ CFU/g. The percentage of positive birds in the ingluvium culture in the positive control group was 50% (*n* = 11/22), and in the cecal contents, 59.09% (*n* = 13/22), with birds demonstrating counts ranging from 1.1 × 10^3^ CFU/g to 3 × 10^6^ CFU/g of cecum. In the group treated with lysozyme, the percentage of positive birds identified was 26.08% (*n* = 6/23), with counts ranging from 2 × 10^4^ CFU/g to 1 × 10^6^ CFU/g of feces.

A Kruskal–Wallis test, followed by Dunn’s post hoc test, was applied to assess the differences among groups. Statistically significant differences (*p* < 0.001) were observed in both ileal and cecal bacterial counts, with the lysozyme-treated group showing a significant reduction compared to the positive control.

## 4. Discussion

The increase in poultry-related MDR *Salmonella* serotypes has been considered a major public health concern in recent years due to the clinical risks to poultry consumers [26,27], as well as the increasing detection of antibiotic resistance in *S*H and *S*M isolates from poultry in Brazil, particularly those carrying the *bla*_CMY-2_, *bla*_CTX-M-2_, and *qnrB19* genes [27].

Some serovars are not host-specific and represent one of the greatest challenges in the poultry sector [28,29]. Although these are non-typhoidal types of *Salmonella*, their frequent association with foodborne outbreaks makes them a barrier to the international poultry trade [30].

Campos et al. [31] emphasized the emergence of extended-spectrum cephalosporin-resistant *S*H and *S*M detected in poultry meat imported into the European Union. Our study showed a high frequency (97.7%) of multidrug-resistant *Salmonella* spp. isolated from the fecal samples of broiler chickens between 2017 and 2022. This finding is similar to that of the study of Perin et al. [32], which reported a prevalence of 85.7% of MDR *Salmonella* in chicken meat in Brazil, which must be considered against the overall national prevalence of 50% *Salmonella* spp. in broiler carcasses at slaughterhouses [33].

In the study by Perin et al. [32], the authors highlighted the presence of extended-spectrum beta-lactamase (ESBL)-producing *Salmonella* spp., which aligns with our findings of SH and SM isolated, showing high MICs for antimicrobial classes used in human medicine. *S*H strains presented a broader resistance profile, including classes of critical and high-priority drugs, such as third-generation cephalosporins and fluoroquinolones. Campos et al. [31] highlighted that ST15 *S*H and ST548 *S*M carried *bla*_CMY-2_ genes in the epidemic plasmids IncA/C and Incl1. Some resistome studies have shown the presence of *bla*_CMY-2_, *bla*_CTX-M-2_, *bla*_CTX-M-8_, *bla*_TEM_, and *bla*_SHV_ and *qnrB19, qnrB5*, and *fosA7* in *S*H and *S*M isolated in some Brazilian states [5,31,33,34]. These findings underscore the urgent need for continuous surveillance in the poultry production chain and the search for natural alternatives, such as enzymes, to reduce dependence on synthetic antimicrobials. The detection of colistin-resistant strains is particularly worrisome, given that colistin is often considered a last-line therapeutic option for carbapenemase-producing bacteria [34].

In recent years, the search for alternatives to antimicrobial use has increased due to the bacterial resistance acquired by pathogens. Greater acceptance of prebiotics, probiotics, essential oils, enzymes, and organic acids has been observed to reduce antibiotic use [35].

The literature reports that lysozyme’s antimicrobial activity is quite effective against *Micrococcus luteus*, *Bacillus stearothermophilus*, and *Clostridium tyrobutyricum* [36]. However, for Gram-negative bacteria, the effect is limited due to their cell wall composition. Davidson et al. [37] reported the inhibition of several microorganisms but highlighted that *S.* Typhimurium is an organism frequently not lysed or inhibited by lysozymes.

The hypothesis of this study was to verify whether lysozyme could act in the reduction in or inactivation of *S*H and *S*M serovars. The microdilution tests revealed that 86.3% of *Salmonella* strains of the Heidelberg and Minnesota serovars had their growth inhibited at concentrations of 15 ppm. However, some *S*H and *S*M strains were resistant to concentrations of 250 to 2000 ppm.

Lysozyme resistance has been described in *Staphylococcus aureus*, *Neisseria gonorrheae*, and *Proteus mirabilis*. The mechanisms of resistance to lysozyme have not yet been fully elucidated, but alterations in the acetylation of carbon 6 of N-acetylmuramic acid are implicated in the reduction or inactivation of muramidase activity [38]. In *Enterococcus faecalis*, lysozyme resistance is also associated with the presence of genes encoding metalloproteases [39]. A third resistance mechanism involves alterations in cell permeability induced by changes in the cationic charge of the cell wall, conferring cross-resistance between polymyxin and lysozyme. In this mechanism, phosphoethanolamine transferase adds positively charged phosphoethanolamine to the lipid A of lipopolysaccharide (LPS). The change in the charge of the bacterial wall repels cationic molecules, reducing susceptibility to lysozyme in *Escherichia coli* strains carrying the *mcr-1* gene (which encodes colistin resistance) by approximately 5- to 20-fold [40]. In this study, two isolates of *S*H with a colistin-resistant phenotype were identified; they showed resistance to 2000 ppm exposure of lysozyme, corroborating the results found by Gogry et al. [40]. Colistin resistance contributes to changes in the outer membrane surface of Gram-negative bacteria, reducing the bactericidal effect of lysozyme.

Only six (9.67%) *Salmonella* spp. Isolates were resistant to lysozyme in the microdilution test, with the highest concentration tested being 2000 ppm. However, many isolates (41.93%) were sensitive to a lower concentration of 15 ppm. The MIC90 was 500 ppm for *Salmonella* spp., but *S*H was more sensitive to lysozyme, with an MIC90 of 250 ppm. These values suggest that the use of lysozyme on poultry farms may be a viable alternative to antibiotics, especially in outbreaks associated with *S*H.

To date, this is the first report demonstrating in vitro bactericidal activity of lysozyme against *S*H and *S*M strains. However, the commercial viability of the product depends on its in vivo action, considering that the intestine is a complex environment with a diverse microbiota [41].

For the in vivo evaluation, an MDR strain of *S*H with complete genome sequencing was selected. This strain was chosen due to the frequency of the serovar and the higher rates of antimicrobial resistance to critical classes of antimicrobials shared with human medicine (third-generation cephalosporins, quinolones, and fosfomycin). The dissemination of *S*H is supported by its high ability to colonize the intestines of chickens, adapt to the environment of farms and slaughterhouses, and form biofilms, which favor resistance to long periods outside the host [42].

Although variations in isolation frequencies were observed across the evaluation time points, no statistically significant differences were detected using the chi-square test with Yates’ correction. Due to the limited sample size and the challenge of establishing clear statistical differences, the data were further analyzed using a non-parametric ANOVA. This analysis revealed significant differences between the treatment group, negative control, and positive control.

The in vivo results closely reflect the challenges faced in commercial poultry production, particularly the issue of flock recolonization associated with the reuse of contaminated substrates across multiple production cycles. Effective *Salmonella* control may require a multifactorial approach in the field, combining lysozyme with other compounds such as probiotics and organic acids. Sabo et al. [35] demonstrated the potential of bacteriocins to inhibit *S. Heidelberg* under in vitro conditions, although these findings have not yet been validated in vivo.

The quantitative analysis conducted in this study demonstrated a significant reduction in bacterial counts in both the ingluvium and ceca of birds from the lysozyme-treated group compared to the positive control group. This reduction may contribute to minimizing cross-contamination during poultry processing, particularly during evisceration and carcass chilling—critical stages in which *Salmonella* transmission commonly occurs.

During the in vivo experiment, *Salmonella* infection was observed from the second day of inoculation, except in the negative control group. Administration of lysozyme at a dose of 1000 ppm demonstrated a significant reduction in the number of infected animals after 21 days.

The administration of lysozyme followed by lactic acid bacteria may represent a promising alternative with potential synergistic effects, leading to improved outcomes. This effect may be attributed to the presence of *Salmonella* in the gastrointestinal tract under conditions where *Lactobacillus* spp. is also present, as these bacteria contribute to pH reduction and modulation of the intestinal microbiota [43]. Such changes create an environment conducive to increased bacterial concentration in the ceca [44], which is considered one of the main reservoirs for *Salmonella* spp. in poultry [45].

Several factors can influence the susceptibility of birds to *Salmonella* infection, including bird age, immune system maturation, diversity of gut microbiota, type of diet, the physical characteristics of the feed particles, and the nature of the bedding material used in poultry houses [46,47,48,49].

The use of lysozyme has already demonstrated benefits in reducing necrotic enteritis [50]. In the present study, lysozyme administration contributed to the reduction in the intestinal colonization of *S. Heidelberg* and *S. Minnesota* in broiler chickens.

## 5. Conclusions

This study reinforces the potential of lysozyme as a natural, innovative strategy for replacing synthetic drugs on farms. It is proven to be an effective antimicrobial alternative in the control of *S*H and *S*M, and demonstrates direct action against the wall of Gram-negative bacteria, culminating in the lysis of the outer membrane and reduction in the pathogen.

## Figures and Tables

**Table 1:** The distribution of the 44 isolates of *Salmonella* spp. according to the MIC values of the antimicrobials tested.

Antibiotic	Numbers of Isolates/MIC µg/mL															CIM 50% µg/mL	CIM 90% µg/mL	% Resistance
	0.06	0.12	0.25	0.5	1	2	4	8	16	32	64	128	256	512	1024			
Nalidixic acid								16	0	0	**27**	**1**				64	64	63.6%
Amoxicilin with clavulanate				11	0	0	6	1	1	0	**25**					64	64	56.8%
Ampicilin					9	2	0	0	0	0	**33**					64	64	75.0%
Azythromycin							6	12	15	**10**	**1**					16	32	25.0%
Ceftiofur			10	0	1	16	0	**17**								2	8	36.6%
Ciprofloxacin	16	0	0	2	**20**	**3**	**2**	**1**								1	2	59.0%
Cloranfenicol							12	26	4	0	**2**					8	16	4.5%
Colistin					36	6	**1**	**1**	0							1	2	4.5%
Fosfomycin								39	2	0	3	0	0	0		8	16	0%
Florfenicol				0	4	0	38	2	0							4	4	0%
Gentamicin				35	0	0	0	1	**5**	**3**	0					0.5	16	18.1%
Marbofloxacin	16	0	1	16	8	2	**1**	0								0.5	1	2.2%
Meropenem			44	0	0	0	0	0								0.25	0.25	0%
Neomycin			0	0	0	0	29	**3**	**12**							4	16	34.0%
Oxytetracycline						1	1	0	0	**42**						32	32	95.4%
Sulfametoxazole with trimetoprim						44	0	0	0	0	0					2	2	0%
Sulfonamides													1	0	43	1024	1024	97.7%

Resistant strains are shown in red.

**Table 2:** The distribution of the 44 isolates of *Salmonella* spp. according to MBC.

Farm	Number of Isolates/MBC ppm								MBC 50%	MBC 90%
	≤15	31	62	125	250	500	1000	>2000		
1—S. Heidelberg	7	1	0	0	0	0	0	1	15	15
2—S. Heidelberg	6	0	0	0	1	0	0	1	15	15
3—S. Heidelberg	11	0	0	0	0	0	0	0	15	15
4—S. Minnesota	14	0	0	0	1	0	1	0	15	15
Total	38	1	0	0	2	0	1	2		
	(86.3%)	(2.2%)	(0%)	(0%)	(4.5%)	(0%)	(2.2%)	(4.5%)		

**Table 3:** The frequency of isolation of *Salmonella* Heidelberg in the feces of broiler chickens from the positive control, negative control, and challenged groups treated with lysozyme at a dose of 1000 ppm.

Bird Age (Days)	T1—Unchallenged Negative Control	T2—Positive Control Challenged	T3—Treatment Group
2	0/12 (0%)	5/12 (41.66%)	2/12 (16.6%)
5	0/12 (0%)	8/12 (66.66%)	4/12 (33.33%)
7	0/12 (0%)	7/12 (58.33%)	4/12 (33.33%)
14	0/12 (0%)	8/12 (66.66%)	7/12 (58.33%)
18	0/12 (0%)	7/12 (58.33%)	3/12 (25%)
21	0/12 (0%)	14/22 (63.63%)	6/23 (26.08%)

**Table 4:** Descriptive statistics of *S.* Heidelberg isolation results by treatment group with number of animals per category (N), percentage with 95% confidence interval (CI), and chi-square association, with Yates correction.

Bird Age (Days)	Isolation	N	Positive Control % (IC95%)	N	Treatment % (IC95%)	*p*-Value
2	Negative	7	41.18 (20.68–64.41)	10	58.82 (35.59–79.32)	0.369
	Positive	5	71.43 (35.23–93.53)	2	28.57 (6.47–64.77)	
5	Negative	4	33.33 (12.45–61.24)	8	66.67 (38.76–87.55)	0.221
	Positive	8	66.67 (38.76–87.55)	4	33.33 (12.45–61.24)	
7	Negative	5	38.46 (16.47–65)	8	61.54 (35–83.53)	0.413
	Positive	7	63.64 (34.8–86.27)	4	36.36 (13.73–65.2)	
14	Negative	4	44.44 (17.3–74.59)	5	55.56 (25.41–82.7)	1.000
	Positive	8	53.33 (29.39–76.12)	7	46.67 (23.88–70.61)	
18	Negative	5	35.71 (15.15–61.55)	9	64.29 (38.45–84.85)	0.214
	Positive	7	70 (39.42–90.73)	3	30 (9.27–60.58)	
21	Negative	8	32 (16.44–51.46)	17	68 (48.54–83.56)	0.025
	Positive	14	70 (48.28–86.39)	6	30 (13.61–51.72)	

**Table 5:** The results of the ANOVA-like analysis (non-parametric ANOVA) regarding the frequency of positivity by treatment group and day of analysis.

ANOVA-like Analysis	*p*-Value
Group	<0.001
Positive control–Negative control	0.000
Positive control–Treatment	0.008
Negative control–Treatment	0.000
Time	0.196
Positive control–Negative control	0.743
Positive control–Treatment	0.196
Negative control–Treatment	0.203
Group–Time	0.728
Positive control–Negative control	0.743
Positive control–Treatment	0.878
Negative control–Treatment	0.203

**Table 6:** The counts of colony-forming units in the ingluvium of chickens in positive control and 1000 ppm lysozyme-treated groups, slaughtered at 21 days.

	Ingluvium						Cecum					
Bird	Positive Control			Treatment			Positive Control			Treatment		
1	1 × 10^3^	1 × 10^3^	1 × 10^2^	-	-	-	3 × 10^6^	2 × 10^5^	1 × 10^6^	-	2 × 10^4^	
2	1 × 10^3^	1 × 10^2^	1 × 10^2^	-	-	-	1.5 × 10^5^	1.4 × 10^5^	1 × 10^5^	-	1 × 10^5^	-
3	3 × 10^2^	1 × 10^3^	-	-	-	-	1.6 × 10^5^	9 × 10^4^	-	2 × 10^4^	-	-
4	1 × 10^5^	1 × 10^2^	-	-	-	-	2 × 10^5^	3 × 10^4^	-	-	-	-
5	4 × 10^2^	-	-	-	-	-	3.6 × 10^5^	1 × 10^5^	-	-	3 × 10^5^	-
6	-	-	-	-	-	-	1.1 × 10^3^	1 × 10^5^	-	-	-	-
7	-	-	-	-	-	-	-	-	-	1 × 10^5^	1 × 10^5^	-
8	*	-	*	-	-	-	-	-	-	*	-	-

(*)—dead bird; (-)—negative bird.

## Data Availability

Data supporting reported results can be found at https://www.teses.usp.br/teses/disponiveis/10/10133/tde-27072023-170502/pt-br.php, accessed on 15 December 2025.

## References

[1] Galán-Relaño Á., Valero Díaz A., Huerta Lorenzo B., Gómez-Gascón L., Mena Rodríguez M.Á., Carrasco Jiménez E., Pérez Rodríguez F., Astorga Márquez R.J. (2023). *Salmonella* and Salmonellosis: An Update on Public Health Implications and Control Strategies. Animals.

[2] Wang Y., Liu Y., Lyu N., Li Z., Ma S., Cao D., Pan Y., Hu Y., Huang H., Gao G.F. (2022). The temporal dynamics of antimicrobial-resistant *Salmonella enterica* and predominant serovars in China. Natl. Sci. Rev..

[3] Wang Y., Xu X., Jia S., Qu M., Pei Y., Qiu S., Zhang J., Liu Y., Ma S., Lyu N. (2025). A global atlas and drivers of antimicrobial resistance in Salmonella during 1900–2023. Nat. Commun..

[4] Ma Y., Chen P., Mo Y., Xiao Y. (2024). WHO revised bacterial priority pathogens list to encourage global actions to combat AMR. Hlife.

[5] Alikhan N.F., Moreno L.Z., Castellanos L.R., Chattaway M.A., McLauchlin J., Lodge M., O’Grady J., Zamudio R., Doughty E., Petrovska L. (2022). Dynamics of Salmonella enterica and antimicrobial resistance in the Brazilian poultry industry and global impacts on public health. PLoS Genet..

[6] El-Shall N.A., Awad A.M., El-Hack M.E.A., Naiel M.A.E., Othman S.I., Allam A.A., Sedeik M.E. (2019). The Simultaneous Administration of a Probiotic or Prebiotic with Live *Salmonella* Vaccine Improves Growth Performance and Reduces Fecal Shedding of the Bacterium in *Salmonella*-Challenged Broilers. Animals.

[7] Souza A.I.S., Saraiva M.M.S., Casas M.R.T., Oliveira G.M., Cardozo M.V., Benevides V.P., Barbosa F.O., Freitas Neto O.C., Almeida A.M., Berchieri Junior A. (2020). High Occurrence of β-Lactamase-Producing *Salmonella heidelberg* from Poultry Origin. PLoS ONE.

[8] Sahoo N., Kumar P., Bhusan B., Bhattacharya T., Dayal S., Sahoo M. (2012). Lysozyme in Livestock: A Guide to Selection for Disease Resistance: A Review. J. Anim. Sci. Adv..

[9] Gong M. (2014). Efficacy of Lysozyme as an Alternative to Antibiotics for Broiler Chickens. Master’s Thesis.

[10] Losso J.N., Nakai S., Charter E.A., Naidu A.S. (2000). Lysozyme. Natural Food Antimicrobial Systems.

[11] Ellison R.T., Giehl T.J. (1991). Killing of Gram-Negative Bacteria by Lactoferrin and Lysozyme. J. Clin. Investig..

[12] Alvarez J., Sota M., Vivanco A.B., Perales I., Cisterna R., Rementeria A., Garaizar J. (2004). Development of a Multiplex PCR Technique for Detection and Epidemiological Typing of *Salmonella* in Human Clinical Samples. J. Clin. Microbiol..

[13] Salmonella Subcommittee of the Nomenclature Committee of the International Society for Microbiology (1934). The Genus *Salmonella* Lignières, 1900. J. Hyg..

[14] Bankevich A., Nurk S., Antipov D., Gurevich A.A., Dvorkin M., Kulikov A.S., Lesin V.M., Nikolenko S.I., Pham S., Prjibelski A.D. (2012). SPAdes: A New Genome Assembly Algorithm and Its Applications to Single-Cell Sequencing. J. Comput. Biol..

[15] Gurevich A., Saveliev V., Vyahhi N., Tesler G. (2013). QUAST: Quality Assessment Tool for Genome Assemblies. Bioinformatics.

[16] Pornsukarom S., Van Vliet A.H.M., Thakur S. (2018). Whole Genome Sequencing Analysis of Multiple *Salmonella* Serovars Provides Insights into Phylogenetic Relatedness, Antimicrobial Resistance, and Virulence Markers across Humans, Food Animals and Agricultural Environmental Sources. BMC Genom..

[17] Sahl J.W., Lemmer D., Travis J., Schupp J.M., Gillece J.D., Aziz M., Driebe E.M., Drees K.P., Hicks N.D., Williamson C.H.D. (2016). NASP: An Accurate, Rapid Method for the Identification of SNPs in WGS Datasets That Supports Flexible Input and Output Formats. Microb. Genom..

[18] Clinical and Laboratory Standards Institute (CLSI) (2023). Performance Standards for Antimicrobial Susceptibility Testing.

[19] Clinical and Laboratory Standards Institute (CLSI) (2024). VET01 Performance Standards for Antimicrobial Disk and Dilution Susceptibility Tests for Bacteria Isolated from Animals.

[20] National Committee for Clinical Laboratory Standards–NCCLS (2008). Performance Standards for Antimicrobial Disk and Dilution Susceptibility Tests for Bacteria Isolated from Animals: Approved Standard-Third Edition.

[21] Schwarz S., Silley P., Simjee S., Woodford N., van Duijkeren E., Johnson A.P., Gaastra W. (2010). Editorial: Assessing the antimicrobial susceptibility of bacteria obtained from animals. J. Antimicrob. Chemother..

[22] São Paulo (Estado) (1995). Portaria SDA nº 126, de 3 de Novembro de 1995. Normas de Credenciamento e Monitoramento de Laboratórios de Diagnóstico das Salmoneloses Aviárias.

[23] R Core Team (2020). R: A Language and Environment for Statistical Computing.

[24] Brunner E., Domhof S., Langer F. (2002). Non-parametric Analysis of Longitudinal Data in Factorial Experiments.

[25] Noguchi K., Gel Y.R., Brunner E., Konietschke F. (2012). nparLD: An R Software Package for the Non-Parametric Analysis of Longitudinal Data in Factorial Experiments. J. Stat. Softw..

[26] Browne A.J., Chipeta M.G., Fell F.J., Haines-Woodhouse G., Hamadani B.H.K., Kumaran E.A., Aguilar G.R., McManigal B., Andrews J.R., Ashley E.A. (2024). Estimating the subnational prevalence of antimicrobial resistant Salmonella enterica serovars Typhi and Paratyphi A infections in 75 endemic countries, 1990–2019: A modelling study. Lancet Glob. Health.

[27] Saidenberg A.B.S., Franco L.S., Reple J.N., Hounmanou Y.M.G., Casas M.R.T., Cardoso B., Esposito F., Lincopan N., Dalsgaard A., Stegger M. (2023). *Salmonella Heidelberg* and *Salmonella Minnesota* in Brazilian broilers: Genomic characterization of third-generation cephalosporin and fluoroquinolone-resistant strains. Appl. Microbiol. Int..

[28] Gast R.K., Porter R.E., Swayne D.E., Boulianne M., Logue C.M., McDougald L.R., Nair V., Suarez D.L., Wit S., Grimes T., Johnson D. (2020). Salmonella Infections. Diseases of Poultry.

[29] Castro-Vargas R.E., Herrera-Sánchez M.P., Rodríguez-Hernández R., Rondón-Barragán I.S. (2020). Antibiotic resistance in *Salmonella* spp. isolated from poultry: A global overview. Vet. World.

[30] Tellez G., Pixley C., Wolfenden R.E., Layton S.L., Hargis B.M. (2012). Probiotics/direct fed microbials for *Salmonella* control in poultry. Food Res. Int..

[31] Campos J., Mourão J., Silveira L., Saraiva M., Correia C.B., Maçãs A.P., Antunes P. (2018). Imported poultry meat as a source of extended-spectrum cephalosporin-resistant CMY-2-producing *Salmonella Heidelberg* and *Salmonella Minnesota* in the European Union, 2014–2015. Int. J. Antimicrob. Agents.

[32] Perin A.P., Martins B.T.F., Barreiros M.A.B., Yamatogi R.S., Nero L.A., Dos Santos Bersot L. (2020). Occurrence, quantification, pulse types, and antimicrobial susceptibility of *Salmonella* sp. isolated from chicken meat in the state of Paraná, Brazil. Braz. J. Microbiol..

[33] Borges K.A., Cisco I.C., Furian T.Q., Tedesco D.C., Rodrigues L.B., do Nascimento V.P., dos Santos L.R. (2020). Detection and quantification of *Campylobacter* spp. in Brazilian poultry processing plants. J. Infect. Dev. Ctries..

[34] Monte D.F., Lincopan N., Berman H., Cerdeira L., Keelara S., Thakur S., Landgraf M. (2019). Genomic features of high-priority *Salmonella enterica* serovars circulating in the food production chain, Brazil, 2000–2016. Sci. Rep..

[35] Sabo S.S., Mendes A.M., Araújo E.D., Muradian L.B.A., Makiyama E.N., Leblanc J.G., Borelli P., Fock R.A., Knöbl T., Oliveira R.P.S. (2020). Bioprospecting of probiotics with antimicrobial activities against *Salmonella heidelberg* and that produce B-complex vitamins as potential supplements in poultry nutrition. Sci. Rep..

[36] Silva S.G.S. (2019). Clonagem, Expressão e Caracterização de Lisozima de *Anopheles darlingi* em *Pichia pastoris*. Ph.D. Thesis.

[37] Davidson P.M., Sofos J.N., Branen A.L. (2005). Antimicrobials in Food.

[38] Herbert S., Bera A., Nerz C., Kraus D., Peschel A., Goerke C., Meehl M., Cheung A., Götz F. (2007). Molecular basis of resistance to muramidase and cationic antimicrobial peptide activity of lysozyme in staphylococci. PLoS Pathog..

[39] Rouchon C.N., Weinstein A.J., Hutchison C.A., Zubair-Nizami Z.B., Kohler P.L., Frank K.L. (2022). Disruption of the *tagF* orthologue in the *epa* locus variable region of *Enterococcus faecalis* causes cell surface changes and suppresses an *eep*-dependent lysozyme resistance phenotype. J. Bacteriol..

[40] Gogry F.A., Siddiqui M.T., Sultan I., Husain F.M., Al-Kheraif A.A., Ali A., Haq Q.M.R. (2022). Colistin interaction and surface changes associated with mcr-1 conferred plasmid mediated resistance in *E. coli* and *A. veronii* strains. Pharmaceutics.

[41] El-Saadony M.T., Salem H.M., El-Tahan A.M., El-Mageed T.A.A., Soliman S.M., Khafaga A.F., Swelum A.A., Ahmed A.E., Alshammari F.A., El-Hack M.E.A. (2022). The control of poultry salmonellosis using organic agents: An updated overview. Poult. Sci..

[42] Hofer E., Da Silva Filho S.J., Dos Reis E.M.F. (1997). Prevalência de sorovares de *Salmonella* isolados de aves no Brasil. Pesqui. Vet. Bras..

[43] Fonseca B.B., Beletti M.E., Silva M.S., Silva P.L., Duarte I.N., Roossi D.A. (2010). Microbiota of the cecum, ileum morphometry, pH of the crop and performance of broiler chickens supplemented with probiotics. Rev. Bras. Zootec..

[44] Danzeisen J.L., Kim H.B., Isaacson R.E., Tu Z.J., Johnson T.J. (2011). Modulations of the chicken cecal microbiome and metagenome in response to anticoccidial and growth promoter treatment. PLoS ONE.

[45] Mohd Shaufi M.A., Sieo A.C.C., Chong C.W., Gan H.M., Ho Y.W. (2015). Deciphering chicken gut microbial dynamics based on high-throughput 16S rRNA metagenomics analyses. Gut Pathog..

[46] Apajalahti J. (2005). Comparative gut microflora, metabolic challenges, and potential opportunities. Biology of Growing Animals.

[47] Fanelli M.J., Sadler W.W., Franti C.E., Brownell J.R. (1971). Localization of *Salmonellae* within the intestinal tract of chickens. Avian Dis..

[48] Ribeiro A.R., Kellermann A., Santos L.R., Nascimento V.P. (2008). Antimicrobial resistance in *Salmonella Enteritidis* isolated from clinical and environmental broiler chickens and breeder broilers. Arq. Bras. Med. Vet. Zootec..

[49] Pan D., Yu Z. (2014). Intestinal microbiome of poultry and its interaction with host and diet. Gut Microbes.

[50] Liu D., Guo Y., Wang Z., Yuan J. (2010). Exogenous lysozyme influences *Clostridium perfringens* colonization and intestinal barrier function in broiler chickens. Avian Pathol..

